# Identification of Differentially Expressed lncRNAs in a CpG ODN-Activated Macrophage

**DOI:** 10.1155/2020/1407654

**Published:** 2020-06-13

**Authors:** Ying Zheng, Xi Luo, Zailong Qin, Zhiguang Zhou

**Affiliations:** ^1^Center for Medical Research, The Second Xiangya Hospital, Central South University, Changsha, Hunan 410011, China; ^2^Department of Metabolism & Endocrinology, The Second Xiangya Hospital, Central South University, Changsha, Hunan 410011, China; ^3^Key Laboratory of Diabetes Immunology, Central South University, Ministry of Education; National Clinical Research Center for Metabolic Diseases, Changsha, Hunan 410011, China; ^4^Genetic and Metabolic Central Laboratory, Maternal and Child Health Hospital of Guangxi, Nanning, Guangxi 530003, China

## Abstract

A macrophage is an important component of innate immunity which can be activated by infection. A series of inflammatory cytokines are produced and released to eliminate pathogens. CpG DNA is an immune stimulator recognized by TLR9, subsequently inducing inflammatory responses in macrophages. Long noncoding RNA (lncRNA) is a novel class of noncoding RNA, whose length is more than 200 nt, but without protein-coding capacity. lncRNAs are involved in many physiological and pathological processes, including inflammatory responses. In our study, a lncRNA microarray assay was performed to identify differentially expressed lncRNAs and mRNAs in RAW264.7 cells at different time points following CpG ODN stimulation. The results revealed that expression levels of 734 lncRNAs and 734 mRNAs were altered at all time points. Gene Ontology (GO) and Kyoto Encyclopedia of Genes and Genomes (KEGG) biological pathway analyses were performed to predict the functions of dysregulated genes. Coexpression networks of lncRNA-mRNA were constructed based on the correlation analysis between differentially expressed lncRNAs and 10 selected upregulated mRNAs, which have been reported to be involved in CpG DNA-induced inflammatory responses. In addition, we selected 8 dysregulated lncRNAs for further validation by quantitative real-time PCR. The present study provided a systematic perspective on the potential functions of lncRNAs in CpG ODN-induced macrophage activation.

## 1. Introduction

Innate immunity is the first line of host defense against the pathogen threats [1]. A macrophage is an important component of innate immunity and plays a crucial role in the inflammatory responses [2]. Recognition of microbial molecules including lipopeptides, lipopolysaccharides, and DNA by pattern recognition receptors of macrophages such as Toll-like receptors (TLRs) will trigger the intracellular signaling cascades. The process promotes the production of inflammatory cytokines, reactive oxygen, nitrogen species, and antimicrobial peptides for anti-infection. The phagocytosis of macrophages is also enhanced to remove the pathogen [2, 3]. However, excessive inflammation in macrophages can cause host damage and even lead to chronic inflammatory diseases, such as obesity, cardiovascular disease, inflammatory bowel disease, and cancer [3]. Thus, it is required for us to better understand the regulatory mechanisms that limit the excessive inflammatory mediators in macrophages.

A great majority of the human and mouse genome is transcribed as noncoding RNAs (ncRNAs), among which microRNAs (miRNAs) are a well-known posttranscriptional regulator of gene expression [4, 5]. Recently, another novel class of ncRNA, long noncoding RNAs (lncRNAs), has also been identified as important regulators of gene expression [6]. lncRNAs are arbitrarily defined as having 200 or more nucleotides to discriminate them from small noncoding RNAs (sncRNAs). On the basis of their genomic localization relative to protein-coding genes, lncRNAs are further categorized as intergenic, overlapping, intronic, and exonic. lncRNAs can control gene expression at the levels of epigenetic control, transcription, RNA processing, and translation [6, 7]. lncRNAs play a significant role in many physiological and pathological processes [6, 7]. In recent years, a series of lncRNAs have been identified in inflammatory responses, and their functions are also being clarified [8–10]. For instance, lincRNA-Tnfaip3 can act as a coregulator of NF-*κ*B to modulate inflammatory gene transcription in mouse macrophages [11]; lincRNA-Cox2 can promote late inflammatory gene transcription in macrophages through modulating SWI/SNF-mediated chromatin remodeling [12].

Bacterial genomic DNA contains unmethylated cytosine followed by guanine (CpG ODN), which can be recognized by TLR9 in macrophages [2, 3]. Studies have shown that both bacterial CpG DNA and synthetic CpG ODN can induce macrophage activation, with the release of proinflammatory cytokines, such as IL-6, IL-12, and TNF-*α*, the production of NO and iNOS, and macrophage polarization [13, 14]. The CpG ODN-induced macrophage activation is involved with a series of inflammatory molecules and pathways. However, the changes of the lncRNA expression pattern and their roles in CpG ODN-activated macrophages have not been clarified.

In the present study, we employed a lncRNA microarray-based profiling assay to detect changes of lncRNAs at different stages of CpG ODN-induced macrophage activation. Gene Ontology (GO) and Kyoto Encyclopedia of Genes and Genomes (KEGG) pathway analyses were performed based on the function of mRNAs. Coexpression and network potential targeting relationship were constructed according to the microarray results and bioinformatics predictions. Our findings revealed the involvement of lncRNAs in the process of CpG ODN-induced macrophage activation.

## 2. Materials and Methods

### 2.1. Cell Culture and Reagents

The mouse macrophage cell line RAW264.7 was cultured in Dulbecco's modified Eagles' medium (DMEM) (Gibco Laboratories, Grand Island, NY, USA) supplemented with 10% fetal bovine serum (FBS) (HyClone, Logan, UT, USA), at 37°C in a humidified atmosphere of 5% CO_2_. The synthetic active CpG ODN (CpG ODN 1826, 5′TCCATGACGTTCCTGACGTT 3′) was purchased from InvivoGen (San Diego, CA, USA). The RAW264.7 cells were treated with 1 *μ*M ODN1826. After 4 h, 8 h, or 16 h, the cells were harvested for RNA isolation.

### 2.2. RNA Extraction and q-PCR

Total RNA from the macrophage cells was extracted by using TRIzol reagents (Thermo Fisher, Waltham, MA, USA), and cDNA was synthesized from 1 *μ*g of total RNA by using a reverse reaction kit, according to the manufacturer's instructions (Promega, Madison, WI, USA). The quantitative real-time PCR analyses for TNF-*α*, IL-6, and IL-1*β* mRNA expression or lncRNA expression were performed in a Roche qRT-PCR detection system (Roche, Basel, Switzerland). *β*-Actin was used as an internal control. The mRNA or lncRNA expression level was calculated using the 2^−ΔΔCt^ method. We compared all other groups with the average ΔCt value of the control group in one PCR experiment and then used 2^−ΔΔCt^ for data analysis. All the results are the average ratios of three different independent experiments. Then, the same group in the different experiment was averaged and further calculated. The primer sequences for qRT-PCR are listed in Table 1.

### 2.3. Measurement of Cytokine Levels by ELISA

The cell-free supernatants were harvested at indicated times. TNF-*α*, IL-1*β*, and IL-6 levels in the supernatants were assessed by ELISA according to the manufacturer's protocol (Cusabio, Wuhan, China).

### 2.4. NO Quantification

The production of NO was determined by detecting the quantity of nitrite in the supernatant from the cells cultured by the Griess method, using a standard curve constructed with nitrite ranging from 5 to 40 *μ*M.

### 2.5. Western Blot

The cells were lysed by 1 × SDS lysis buffer containing protease inhibitors. The proteins were subjected to electrophoresis on 8% SDS/PAGE gels and then transferred into the polyvinylidene difluoride (PVDF) membrane. Subsequently, they were determined with antibodies against iNOS (Cell Signaling Technology, Boston, MA, USA) and GAPDH (Millipore, Bedford, MA, USA).

### 2.6. lncRNA Microarray Analysis

Total RNA was extracted from RAW264.7 cells stimulated with ODN1826 (1 *μ*M) at different time points according to the study design. The RNA quantity and quality were assessed by NanoDrop. And RNA integrity was detected by capillary electrophoresis using an RNA 6000 Nano Lab-on-a-Chip kit and the Bioanalyzer 2100. The GPL22782-Agilent-074512 Mouse LncRNA Microarray V4.0 was adopted for the detection of lncRNA and mRNA expression, and 40,825 lncRNAs and 30,680 mRNAs were detected. The lncRNA microarray was conducted by CapitalBio Technology (Beijing, China).

### 2.7. Differential lncRNA and mRNA Screening and Clustering Analysis

The raw data of each array result was normalized and then subjected to GeneSpring software (v. 13.0, Agilent). Differentially expressed lncRNA and mRNA were screened with a *p* value < 0.05 and fold change > 2.0. Cluster software (v. 3.0) was employed to analyze differentially expressed lncRNAs and mRNAs. The normalized expression level of each RNA type was further analyzed with hierarchical clustering (HCL). The results were presented by using TreeView software (v. 1.5). The color green-black represents low expression, while red represents high expression. Difference integration analysis (Venn analysis) was also done. The common elements between the stimulated cells were determined by Venn analysis. Often up- and downregulated RNAs were shown in pies with different colors.

### 2.8. GO and Pathway Analyses

Differentially expressed mRNAs were selected for target prediction. GO analysis and pathway analysis were used to determine the roles of these dysregulated mRNAs in biological pathways or GO terms. We uploaded all differentially expressed mRNAs at the different time points into the Database for Annotation, Visualization and Integrated Discovery (DAVID) for annotation and functional analysis, including gene set enrichment analysis and mapping gene sets to the KEGG pathway. GO terms with *p* value less than 0.05 were selected. The top 10 enriched GO terms associated with upregulated or downregulated mRNAs were presented. KEGG pathway analysis was also performed to determine the involvement of differentially expressed genes in different biological pathways.

### 2.9. lncRNA-mRNA Coexpression Network

To predict the functions of differentially expressed lncRNAs, we constructed the lncRNA-mRNA coexpression network. We chose 10 significantly upregulated genes involved in inflammatory signaling pathways to build the CNC network based on the degree of correlation. Pearson's correlation coefficient value was calculated for lncRNA-mRNA pairs, and strong correlated pairs (0.99 or greater) were included (either positive or negative) in the coexpression network. A *p* value < 0.05 was considered statistically significant. We drew the coexpression networks using Cytoscape software (v. 3.2.1).

### 2.10. Statistics

All statistical analyses were conducted by using the SPSS 16.0 software (Chicago, IL, USA) and GraphPad Prism 5.0. Results are expressed as mean ± SD from at least three experiments. Student's *t*-test was used to determine the significance of difference between different groups. *p* < 0.05 was considered significant.

## 3. Results

### 3.1. CpG ODN Induced Inflammatory Responses in Macrophages

**Figure 1:**
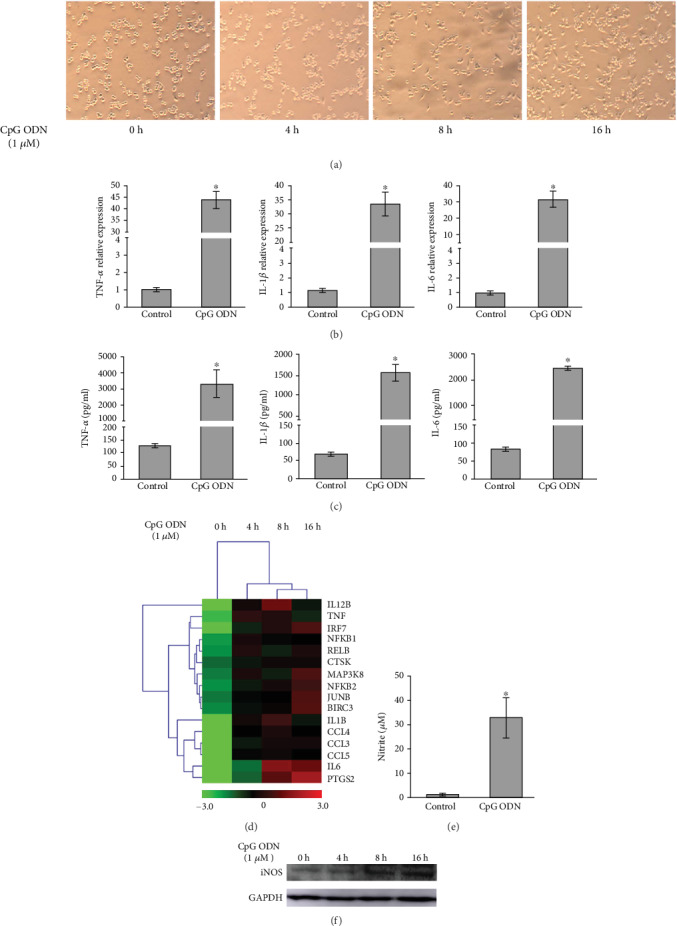
CpG ODN induced macrophage activation. (a) Representative phase-contrast images of RAW264.7 cells stimulated with CpG ODN1826 (1 *μ*M) for 0 h, 4 h, 8 h, and 16 h. (b, c) The RAW264.7 cells were treated with CpG ODN1826 (1 *μ*M) for 16 h. And then, the mRNA and protein expression levels of TNF-*α*, IL-1*β*, and IL-8 were determined by qRT-PCR (b) and ELISA (c). (d) Inflammation-related gene expression levels were altered in the process of CpG ODN1826-induced macrophage activation from 0 h to 16 h. (e) Nitrite was determined in the supernatant of RAW264.7 cells stimulated with CpG ODN1826 for 16 h. (f) iNOS protein levels were detected by Western blot in RAW264.7 cells after stimulation with CpG ODN1826 for 16 h. ^∗^*p* < 0.05.

CpG DNA can be recognized by TLR9 in immune cells, consequently inducing the release of proinflammatory cytokines, as well as the production of NO and iNOS. In our study, we firstly observed inflammatory responses in CpG ODN-activated macrophages. The mouse macrophage cell line RAW264.7 was stimulated with an active ODN type, CpG ODN1826. Then, at the different time points of 4 h, 8 h, and 16 h after the treatment of ODN1826, we observed the morphological changes of the cells (Figure 1(a)). In addition, we detected the mRNA and protein levels of three critical proinflammatory cytokines, TNF-*α*, IL-1*β*, and IL-6, by qRT-PCR and ELISA, respectively. As shown in Figures 1(b) and 1(c), after the treatment of ODN1826, TNF-*α*, IL-1*β*, and IL-6 expression increased. The expression of lncRNAs and mRNAs was detected by GPL22782-Agilent-074512 Mouse LncRNA Microarray v4.0, which includes 40,825 lncRNAs and 30,680 mRNAs. The gene microarray analysis for CpG ODN-activated macrophages was consistent with TLR9 signaling activation, which showed that most inflammatory related-gene expression was upregulated (Figure 1(d)). Meanwhile, we observed the effects of ODN1826 on M1 inflammatory responses, as manifested by the significantly increased NO and iNOS. RAW264.7 macrophages were treated with ODN1826. The production of NO and iNOS in the macrophages was determined. In RAW264.7 macrophages, ODN1826 stimulation could promote the production of NO and iNOS (Figures 1(e) and 1(f)). These data indicated that ODN1826 could induce a remarkably stronger inflammatory response.

### 3.2. Changed Expression Profiles of lncRNAs and mRNAs in CpG ODN-Activated Macrophages

**Figure 2:**
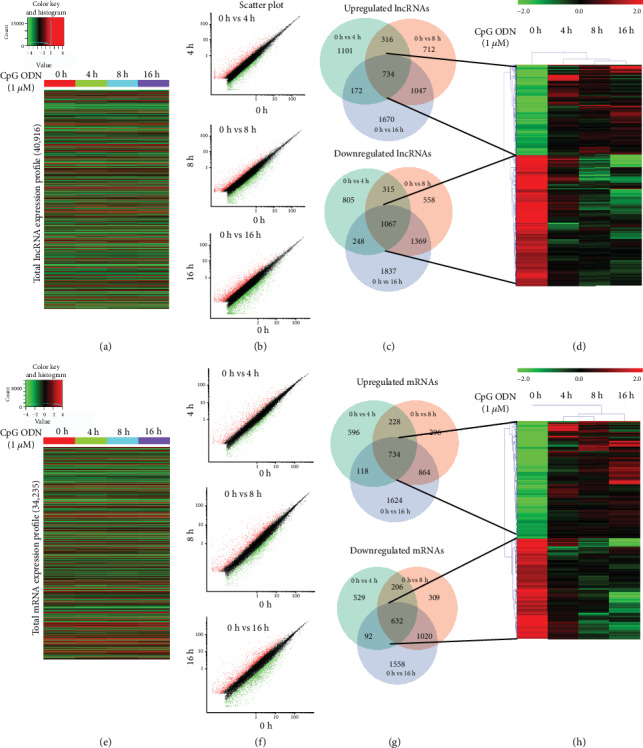
Changed expression profiles of lncRNAs and mRNAs during CpG ODN-induced macrophage activation. (a) The heat map of all lncRNA expression at different time points during CpG ODN-induced macrophage activation from microarray data. (b) Scatter plots showing differentially expressed lncRNAs between macrophages with the stimulation with CpG ODN at 0 h and activated macrophages at different time points (4 h, 8 h, and 16 h). (c) Often differentially expressed lncRNAs between macrophages with the stimulation with CpG ODN at 0 h and activated macrophages at different time points (4 h, 8 h, and 16 h). (d) Hierarchical clustering showing often upregulated and downregulated lncRNAs among the four groups (macrophages stimulated for 0 h, 4 h, 8 h, and 16 h). (e) The heat map of all mRNA expression at different time points during CpG ODN-induced macrophage activation from microarray data. (f) Scatter plots showing differentially expressed mRNAs between macrophages with the stimulation with CpG ODN at 0 h and activated macrophages at different time points (4 h, 8 h, and 16 h). (g) Often differentially expressed mRNAs between macrophages with the stimulation with CpG ODN at 0 h and activated macrophages at different time points (4 h, 8 h, and 16 h). (h) Hierarchical clustering showing often up- and downregulated mRNAs among the four groups (macrophages stimulated for 0 h, 4 h, 8 h, and 16 h).

Total RNA from CpG ODN-treated macrophages at different time points was extracted. The CapitalBio Technology mouse lncRNA microarray v4.0 was applied for the profiling analysis of mouse lncRNAs and mRNAs. In total, 40,825 lncRNAs and 30,680 mRNAs detected are presented in Figures 2(a) and 2(e). Three comparison groups were set in accordance with the different time points following CpG ODN stimulation (0 h vs. 4 h, 0 h vs. 8 h, and 0 h vs. 16 h) (Figures 2(b) and 2(f)). At 4 h after stimulation, 2,323 lncRNAs and 1,676 mRNAs were upregulated, while 2,435 lncRNAs and 1,459 mRNAs were downregulated (Figures 2(c) and 2(g)). At 8 h after stimulation, 2,809 lncRNAs and 2,122 mRNAs were upregulated, while 3,039 lncRNAs and 2,167 mRNAs were downregulated (Figures 2(c) and 2(g)). At 16 h after stimulation, 3,623 lncRNAs and 3,340 mRNAs were upregulated, while 4,521 lncRNAs and 3,302 mRNAs were downregulated (Figures 2(c) and 2(g)). All the differentially expressed lncRNAs and mRNAs were statistically significant (*p* < 0.05) with fold change greater than 2.0. Venn analysis showed that 734 lncRNAs and 734 mRNAs were always upregulated and 1,067 lncRNAs and 632 mRNAs were always downregulated at all time points (Figures 2(c) and 2(g)). A cluster was generated and analyzed with hierarchical clustering (HCL) for the 734 differentially upregulated lncRNAs and 1,067 downregulated lncRNAs (Figure 2(d)). In the same way, a cluster was generated and analyzed with HCL for the 734 upregulated mRNAs and 632 downregulated mRNAs (Figure 2(h)). The information on the data was submitted to the Gene Expression Omnibus, and the accession number is GSE120417.

### 3.3. Validation for the Expression of Significant Transcripts by qRT-PCR

**Figure 3:**
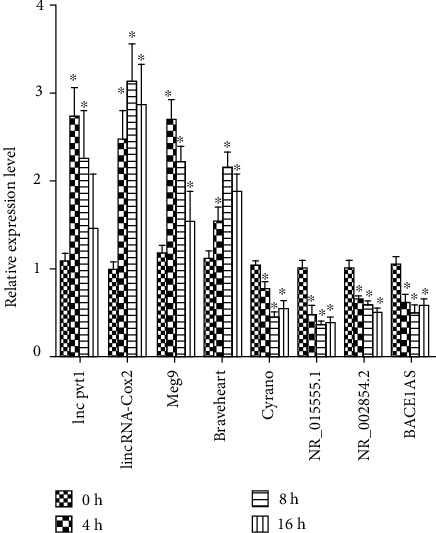
Validation for the expression of significant dysregulated lncRNAs. The RAW264.7 cells were treated with CpG ODN1826, and the RNA was extracted at different time points (0 h, 4 h, 8 h, and 16 h) after stimulation. The relative expression levels of 8 selected lncRNAs were determined by qRT-PCR. Data are presented as mean ± SD. ^∗^*p* < 0.05.

We selected 8 lncRNAs to verify the microarray results by qRT-PCR assays. The results showed that upon the stimulation with CpG ODN, the expression of lncRNA lnc pvt1, lincRNA-Cox2, Meg9, and Braveheart in CpG DNA-stimulated macrophages was upregulated, whereas Cyrano, NR_015555.1, NR_002854.2, and BACE1AS were downregulated (Figure 3). The result is consistent with the microarray assay, which verified the veracity of microarray results. The finding provided evidence that these lncRNAs could be involved in CpG ODN-induced macrophage activation.

### 3.4. Delineation of Gene Ontology (GO) and KEGG Pathway Analyses

**Figure 4:**
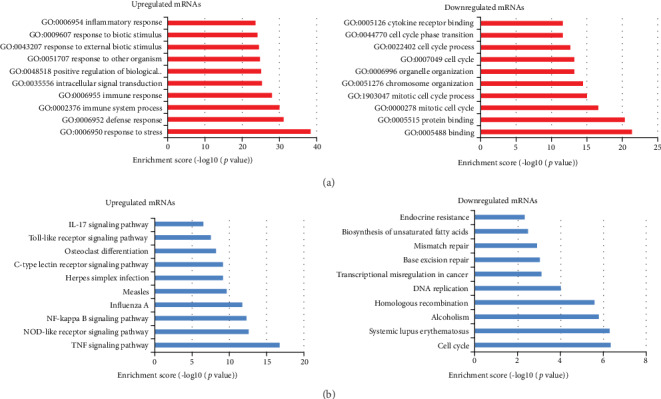
Gene Ontology (GO) and KEGG pathway analyses. (a) GO MF terms for upregulated and downregulated mRNAs were analyzed. Top 10 upregulated and 10 downregulated GO terms ranked by fold enrichment and enrichment score were shown. (b) KEGG pathway enrichment analysis of up- and downregulated mRNAs was performed. Top 10 pathways ranked by the enrichment score were shown.

Next, all differentially expressed mRNAs as described in Figure 2 were further analyzed by DAVID Bioinformatics Resources 6.7. The GO enrichment analysis was conducted mainly on three domains, namely, biological process (BP), cellular component (CC), and molecular function (MF) for upregulated and downregulated mRNAs, respectively. Interestingly, for upregulated mRNAs, the most enriched and meaningful terms belonged to the BP category, most of which were related to immunity, while GO terms associated with downregulated mRNAs were binding, protein binding, mitotic cell cycle, etc. (Figure 4(a)). Moreover, KEGG pathway enrichment analysis was also made. Our data showed 10 pathways associated with upregulated mRNAs and downregulated mRNAs, respectively. Similarly, the top pathways in upregulated protein-coding genes were involved with the TNF signaling pathway, NOD-like receptor signaling pathway, and NF-kappa B signaling pathway (Figure 4(b)). These results revealed that these pathways might be implicated in CpG ODN-induced macrophage activation.

### 3.5. Construction of the lncRNA-mRNA Coexpression Network

**Figure 5:**
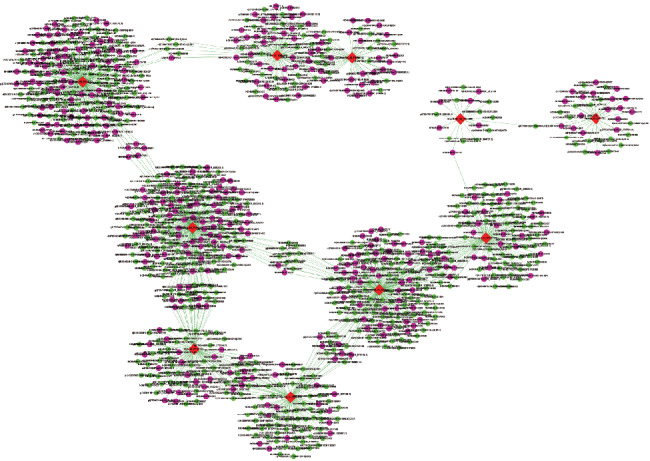
Coexpression network of ten significantly upregulated mRNAs with their associated lncRNAs. The network was based on Pearson's correlation coefficient (the absolute value of PCC ≥ 0.90, *p* value < 0.01, and FDR < 0.01), and solid lines mean positive correlations while dashed lines mean negative correlations. Diamond nodes represent mRNAs, and purple circle nodes represent lncRNAs. Red color and pink color represent upregulation; green color represents downregulation.

Up to now, the roles of most lncRNAs have not been annotated. So the functional prediction of lncRNAs is partially dependent on the coexpressed mRNA function. Herein, we chose 10 significantly upregulated mRNAs to build the CNC network (Figure 5). These 10 mRNAs, including Ctsk, Nfkb2, IL-1*β*, and IL-6 are involved in inflammatory responses and play important roles in the regulation of inflammatory signals. The network is based on Pearson's correlation coefficient (the absolute value of PCC ≥ 0.99, *p* value < 0.01, and FDR < 0.01). From the network, we observed that these important molecules were intimately related to a number of lncRNAs. For example, upregulated NR_033616.1 was positively associated with Ctsk, and lincRNA-Cox2 was positively related to Nfkb2.

## 4. Discussion

Bacterial genomic DNA contains unmethylated CpG DNA, which is a well-known immunostimulator, and triggers innate immunity against infection as well as an adaptive immune response [2, 3]. Furthermore, a short synthetic oligonucleotide containing a CpG motif (CpG ODN) can also function as an agnostic to activate immune signals. For instance, both bacterial DNA and synthetic CpG ODN can induce the release of inflammatory cytokines, including TNF-*α*, IL-1*β*, and IL-6, as well as the production of NO and iNOS [13, 14]. In the present study, we observed the details of CpG ODN-induced inflammatory responses in macrophages. The results showed that CpG ODN1826 stimulation could cause the morphological change of macrophage cells and induce the change of inflammatory gene expression, subsequently promoting the release of inflammatory cytokines and the production of NO and iNOS.

Differing from LPS recognized by TLR4, CpG DNA is recognized by TLR9 in immune cells, including macrophages. Upon CpG DNA stimulation, TLR9 recruits MyD88 that activates p38 and c-Jun, consequently leading to the activation of transcription factors including AP-1 and NF-*κ*B, and the production of inflammatory mediators [13, 14]. CpG ODN-induced inflammatory response is a fairly complex process, which is involved with many molecules, such as inflammatory mediators, transcription factors, and regulatory factors. Long noncoding RNAs (lncRNAs) are a subgroup of noncoding RNAs (ncRNAs) with the length more than 200 nt, but without protein-coding potential [6]. Recently, lncRNAs have been reported to be implicated in inflammatory responses [8, 9]. lncRNA FIRRE regulates inflammatory gene expression through interacting with hnRNPU in macrophages [15]. A natural antisense transcript, AS-IL1*α*, controls inducible transcription of the proinflammatory cytokine IL-1*α* [16]. Mao et al. identified differentially expressed lncRNAs in the process of TLR4 signaling activation in mouse macrophages [17], while another study performed by Huang et al. reported differentially expressed lncRNAs in polarized macrophages followed by the stimulation with IFN-*γ*+LPS or IL-4 [18]. In addition, Dou et al. also identified changed expression profiles of lncRNAs, mRNAs, circRNAs, and miRNAs during osteoclastogenesis [19]. In our study, the expression profiles of lncRNAs and mRNAs in mouse macrophage cells were detected at different time points following CpG ODN1826 stimulation. From the results, we figured out that during the process of CpG ODN-induced macrophage activation, thousands of lncRNAs were differentially expressed compared with the control group. It is very interesting to notice that the expression pattern of lncRNAs was consistent with mRNAs.

The annotation results of the most significant Gene Ontology showed that the top 10 increased biological processes belong to or are associated with immunity. Meanwhile, KEGG pathway analysis for the differentially expressed mRNAs revealed that top 10 pathways associated with upregulated mRNAs were the TNF signaling pathway, NOD-like receptor signaling pathway, NF-kappa B signaling pathway, influenza A, measles, herpes simplex infection, C-type lectin receptor signaling pathway, osteoclast differentiation, Toll-like receptor signaling pathway, and IL-17 signaling pathway, most of which are involved in inflammatory responses.

From the lncRNA-mRNA coexpression network, we found that functional molecules and inflammatory cytokines involved in CpG DNA-induced inflammatory response, including Ctsk, Nfkb2, IL-1*β*, and IL-6, were coexpressed with multiple lncRNAs, which formed a complex network. The CNC analysis result indicated that the change of lncRNA expression might be implicated in CpG ODN-induced macrophage activation by regulating mRNAs. Interestingly in our study, most of the lncRNAs in the coexpression network have not been annotated yet. It will be much worthy to perform further study in exploring the underlying mechanisms of these lncRNAs.

## 5. Conclusion

Innate immunity is the first line of host defense. Despite the benefits of innate immunity, it can be a double-edge sword as excessive inflammation will cause host damage. So it is important to better understand the regulatory mechanisms that control inflammatory responses. Up to date, many molecules and signaling pathways involved in macrophage polarization have been reported [20–24]. Recently, a number of studies in the immune system have provided us accumulating evidence that lncRNAs also play an important role in regulating inflammatory responses. In the present study, we identified a profile of dysregulated lncRNAs that might be involved in CpG ODN-induced macrophage activation. Our data provides a perspective for further functional research of lncRNAs in CpG ODN-induced inflammatory responses and helps to clarify the mechanisms of inflammation.

## Figures and Tables

**Table 1:** qRT-PCR primers used.

Gene or lncRNA	Forward primer	Reverse primer
TNF-*α*	CAGGCGGTGCCTATGTCTC	CGATCACCCCGAAGTTCAGTAG
IL-1*β*	GAAATGCCACCTTTTGACAGTG	TGGATGCTCTCATCAGGACAG
IL-6	CCCAATTTCCAATGCTCTCCT	AGTGGTATAGACAGGTCTGTTGG
lnc pvt1	AGCGTTGACTTAAGAGATGCCA	GATTGCCTCCGGCATGAAGA
lincRNA-Cox2	AGTATGGGATAACCAGCTGAGGT	GAATGCTGAGAGTGGGAGAAATAG
Meg9	AGGCTATCACCATCCCCCTT	TCCTAGACCTTGCCCGATGA
Braveheart	TCTCCTGGAGCCACATCTCT	GCTTTTCCTTAGGCCCAAAC
Cyrano	GAAACATAGGCTGGGACAAT	TGTTACTGGGCTCTGTTT
NR_015555.1	TGGAGGAGCCAGGACTCAAAT	TCCAGAAATCGGGCTGCTTAT
NR_002854.2	GCAGACAGAATTGGGTCGTT	CTCAACTACCGCCTGCAAA
BACE1AS	TCAATGCTAACCTGGGCTACG	TTCCCATCAGGCGCTTACA
*β*-Actin	TGGTGGGAATGGGTCAGAA	TCTCCATGTCGTCCCAGTTG

## Data Availability

All the data except microarray data used to support the findings of this study are included within the article. The microarray data had been deposited in Gene Expression Omnibus (GSE120417).

## Abbreviations

lncRNA: Long noncoding RNA
PPRs: Pattern recognition receptors
TLRs: Toll-like receptors
ncRNAs: Noncoding RNAs
miRNAs: MicroRNAs
sncRNAs: Small noncoding RNA
GO: Gene Ontology
KEGG: Kyoto Encyclopedia of Genes and Genomes.
